# Association between physical activity and metabolic syndrome: a cross sectional survey in adolescents in Ho Chi Minh City, Vietnam

**DOI:** 10.1186/1471-2458-10-141

**Published:** 2010-03-17

**Authors:** Trang HHD Nguyen, Hong K Tang, Patrick Kelly, Hidde P van der Ploeg, Michael J Dibley

**Affiliations:** 1 Department of Community Health, Pham Ngoc Thach University of Medicine, Ho Chi Minh City, Vietnam; 2 The Sydney School of Public Health, Sydney Medical School, University of Sydney, Sydney, NSW 2006, Australia; 3 Cluster for Physical Activity and Health, Sydney School of Public Health, University of Sydney, Sydney, NSW 2006, Australia

## Abstract

**Background:**

The emerging epidemic of overweight/obesity in adolescents in Ho Chi Minh City, Vietnam underlines the importance of studying the metabolic syndrome in Vietnamese adolescents who are becoming progressively more inactive. No study in Vietnam has examined the association of metabolic syndrome with moderate to vigorous physical activity (PA) levels among adolescents. We aimed to examine this association in a sample of urban adolescents from Ho Chi Minh City.

**Methods:**

A cross-sectional assessment was conducted in 2007 on a representative sample of 693 high-school students from urban districts in Ho Chi Minh City. Metabolic syndrome was defined according to the International Diabetes Federation criteria and physical activity was measured with Actigraph accelerometers. The association between physical activity and metabolic syndrome was assessed by using multiple logistic regression models.

**Results:**

Overall 4.6% of the adolescents and 11.8% of the overweight/obese adolescents had metabolic syndrome. Elevated BP was the most common individual component of the metabolic syndrome (21.5%), followed by hypertriglyceridemia (11.1%). After adjusting for other study factors, the odds of metabolic syndrome among youth in the lowest physical activity group (<43 minutes of physical activity/day) were five times higher than those in the highest physical activity group (>103 minutes/day) (AOR = 5.3, 95% CI: 1.5, 19.1). Metabolic syndrome was also positively associated with socioeconomic status (AOR = 9.4, 95% CI: 2.1, 42.4).

**Conclusions:**

A more physically active lifestyle appears to be associated with a lower odds of metabolic syndrome in Vietnamese adolescents. Socio-economic status should be taken into account when planning interventions to prevent adolescent metabolic syndrome.

## Background

Metabolic syndrome is a clustering of cardiovascular disease risk factors that includes glucose intolerance, hypertension, elevated triglycerides, low HDL cholesterol, and obesity [1]. This clustering has been shown to occur not only in adults but also in adolescents [2-8].

This syndrome continues to increase in both developed and developing countries, but has already become a major threat to global public health. It is especially of concern when it affects children and adolescents, as a consequence of increasing rates of childhood obesity and more sedentary lifestyles [9-11]. The prevalence of the metabolic syndrome in children and adolescents is relatively low (4%) when compared to the adult population (24%), except amongst overweight and obese adolescents where the prevalence of the metabolic syndrome has been reported as high as 29% [2,3,12].

Studies in adults and also in adolescents reveal that physical activity (PA) - a modifiable lifestyle factor - is strongly associated with clustering of metabolic syndrome components [13-18]. Recently, it has been shown that there might be an independent, inverse relationship between objectively measured PA and metabolic risk factors in children [14]. It has also been shown that higher levels of physical activity are also positively correlated with insulin sensitivity in adolescents, even in the absence of weight loss [19].

Vietnam is undergoing a socio-economic and nutrition transition especially in large cities, such as Ho Chi Minh City, where lifestyles are becoming more sedentary with lower levels of PA, and diets more energy dense with higher fat content. The nutrition transition has lead to a threefold increase in the prevalence of overweight/obesity over the last five years among adolescents in Ho Chi Minh City [20]. The emergent epidemic of obesity in adolescents makes metabolic syndrome and its sequelae an important condition to study in Vietnamese adolescent populations. This is especially the case because a recent study found that 24.3% of Vietnamese junior high school students were inactive [21].

Although the association of metabolic syndrome with different levels of objectively assessed PA [13-16] and self reported PA [17,18] among adolescents has been reported in other settings [13-18], no study has examined this relationship in Vietnamese youth. This study aimed to examine the association between physical activity and metabolic syndrome in adolescents in Ho Chi Minh City, Vietnam.

## Methods

### Study design

A cross-sectional assessment was conducted in 2007 on a representative sample of high-school students from urban areas of Ho Chi Minh City. These students had been recruited into a cohort study from a sub-sample of students from urban districts identified in a survey in 2004 [22]. The sample size of this study was calculated with insufficient physical activity as the primary outcome. A study in 2004 among adolescents in urban areas of Ho Chi Minh City [21] reported a prevalence of adolescent insufficient activity of 24.3%. It is estimated that this prevalence would change by at least 10%. Using this information and assuming a 95% confidence interval, with a precision of 0.05, and an adjustment for clustering effect of 2.1 (based on a similar study from 2004 [22]) suggests a sample size of 585. Given an average of 35 students per class, this equates to approximately 18 classes from 18 schools selected and a total sample size of 630 students. Multi-stage cluster sampling was used to select these students who were therefore representative of the junior high school students for these districts. The study was approved by the ethics committee for medical research of Pham Ngoc Thach University of Medicine (PNTUM), and the Human Research Ethics Committee of the University of Newcastle, Australia. In addition, the study was approved by the city education department, the school health division at the district level and the headmasters at each of the selected schools. Written consent was obtained from both students and their parents prior to data collection.

### Data collection

Two trained data collectors took the anthropometric measurements of the students using standard methods [23]. Weight was measured without shoes and heavy clothes, using a Tanita electronic scale (Tanita BF 571, Tanita Corporation, Japan) and was recorded to the nearest 100 g. Standing height was measured with a suspended Microtoise tape to the nearest 0.1 cm. Anthropometric standardization exercises were conducted to ensure uniform techniques were used by all data collectors [23].

Body mass index (BMI) was calculated as weight in kilograms divided by the square of height in meters (kg/m^2^). The subjects were classified as overweight/obese by applying the age and sex specific International Obesity Task Force (IOTF) BMI cut off points [24].

Waist circumference was measured to the nearest 0.5 cm with a non-elastic tape applied at a point midway between the lower border of the rib cage and the iliac crest at the end of normal expiration. Smoothed percentiles for waist circumference were constructed from the raw data and were entered onto a spreadsheet separately for boys and girls and imported into the software package LMS [25]. The LMS method enables normalised growth centile standards to be developed, and deals generally with skewness which might be present in the distribution of the circumference measurements. This method assumes that the data can be normalised by using a power transformation, which removes skewness from the data set by extending one tail of the distribution and reducing the other [26]. The maximum power required to obtain normality was calculated for each age group series and the trend was then summarised by a smooth (L) curve. The trends observed for the mean (M), and coefficient of variation (S), were similarly smoothed. These LMS curves contained information to draw any centile curve, and to convert measurements into exact SD scores [26].

Skinfold thickness was measured with a Harpenden caliper at the triceps, sub-scapular, abdominal, and medial-calf skinfold using a standard measurement method [27]. The mean of the two skinfold measurements taken at each site was used, and the sum of these mean skinfold measurements from each of the four sites was calculated.

Determination of pubertal development was assessed by a self-administered confidential questionnaire using diagrams illustrating the five stages of pubertal development. Tanner's stages for (1) male genitalia, (2) male pubic hair, and (3) female breast were used in the pubertal assessment [28]. In addition, the date of the first menstruation was also asked of female students and attainment of adult voice was asked of the male students. Pubertal stage was grouped into pre-pubescent, pubescent and post-pubescent based on the WHO definition [23]. In girls the pubertal stages were classified as follows: pre-pubertal - breast was at stage 1, but they had not attained menarche; pubertal - breast was at least at stage 2 but they had not had the first menstrual period; post-pubertal - breast was at least at stage 2 and had already had the first menstrual period. In boys the pubertal classification was as follows: pre-pubertal - the genitalia were at stage 2 or less and they had not attained adult voice; pubertal - genitalia were at least at stage 3 or above but they still did not have an adult voice; and post-pubertal - genitalia were at least at stage 3 or above, and they had attained an adult voice.

Fasting blood samples of 5 ml were taken in the morning in the clinic room at the subject's school by a trained laboratory technician. On the previous day, the students were instructed to fast for 12 hours before the test, and their adherence to this protocol was confirmed on the morning of the examination before drawing blood. All blood specimens for serum lipids were collected in plain tubes. Plasma for glucose measurements was collected in fluoride/oxalate tubes. They were kept in ice at 0°C before being transported back to the Laboratory of the Diagnostic Center of Ho Chi Minh City for further processing. All assays were performed within 6 hours after collection, and additional aliquots of serum were stored at -70°C. Serum lipid and glucose concentrations were measured and read on an automated analyzer BM/Hitachi 917 analyzer (Roche Diagnostics, Corp. Indianapolis, IN, USA) using reagent kits supplied by the manufacturer of the analyzer. Both the intra-assay and inter-assay coefficients of variation for glucose were 2% at 6.6 mM. Total cholesterol (intra-assay CV 0.8%, inter-assay CV 1.7%), triglyceride (TG) (intra-assay CV 1.5%, inter assay CV 1.8%) were measured using enzymatic calorimetric assays. HDL-C (intra-assay CV 2.9%, intra-assay CV 3.6%) was measured using a homogenous colorimetric assay. This assay does not involve a precipitation step. Direct Low density lipoprotein cholesterol (LDL-C) (Intra-assay CV 0.9%, inter-assay CV 2.0%) was measured using a homogenous turbidimetric assay.

Systolic and diastolic blood pressures (BP) were measured with an automatic oscillometric method (Omron BP monitor) after 15 minutes of rest. The subject was in a seated, relaxed position and recordings were made 2 times at 10 minute intervals. The measurements were taken in millimeters of mercury and the mean value of the two recordings (not varying by more than 5 mm Hg) was calculated.

Physical activity was assessed with Actigraph GT1 M accelerometers (Actigraph, LLC, Fort Walton Beach, FL). The Actigraph GT1 M is a single axis accelerometer that records activity counts, which were stored every minute. Participants wore the accelerometer on the right hip for seven consecutive days, except during sleep and water based activities. Average time spent per day in moderate to vigorous physical activity (≥ 3.0 MET) was calculated using existing age specific cut points for accelerometer activity counts [29]. Only participants who wore the accelerometer for eight or more hours per day on at least four days were included in the analysis.

To assess household economic status, ownership of fourteen different assets (telephone, radio, video cassette player, CD system, DVD player, air conditioner, refrigerator, computer, gas stove, microwave, bicycle, motorbike, car, television) were used to construct a household wealth index using the principal components method to assign a weight for each asset [30]. The index was ranked and divided into quintiles and each household was assigned to one of these wealth index categories. This wealth index was used to rank families by their wealth status.

### Definition of metabolic syndrome

Since no standard definition exists for the metabolic syndrome in adolescents, subjects were classified as having metabolic syndrome according to the International Diabetes Federation (IDF) guidelines [31]. This definition includes waist circumference as a prerequisite and adheres to the adult values for hypertension, glucose intolerance, and dyslipidemia [31].

Metabolic syndrome was defined as adolescents with waist circumference ≥ 90th percentile and two or more of the following components: fasting glucose ≥ 5.6 mmol/L, triglycerides ≥ 1.70 mmol/L, HDL ≤ 1.03 mmol/L, and/or BP ≥ 130 mmHg systolic or ≥ 85 mmHg diastolic [31].

These IDF criteria, with age-specific guidelines to extend their use to children under 16 years of age, have the advantage of using age, sex, and ethnic specific criteria for waist circumference. This may compensate for variations in childhood and allow for comparison of the prevalence of adolescent metabolic syndrome across different ethnic groups. In addition, the IDF guidelines include abdominal obesity as a mandatory criterion, since abdominal obesity is known to be a strong predictor of cardiovascular risk factors [32,33]. Therefore, these criteria are a useful and unified tool for identifying adolescents with metabolic syndrome.

### Statistical analysis

All data were analyzed using STATA 10.0 (StataCorp, 2005). Mean and 95% CI for age, weight, and components of the metabolic syndrome were calculated by sex and age. Daily time for moderate and vigorous activity in minutes were calculated and then log-transformed to correct for the skewness of the distributions. Gender and age group differences in physical activity variables were examined using t-tests and ANOVA. Since data was not normally distributed, time spent on moderate to vigorous physical activities between two groups was compared by Mann-Whitney rank sum test. The "survey commands" were used to account for the multi-stage cluster sampling design. Multiple logistic regression was used to assess associations between quintiles of physical activity as well as other covariates (age, sex, pubertal mature, mother's education, socio-economic status) and metabolic syndrome. Variables with a univariate p-value < 0.25 were entered into the multivariate model. Stepwise backward elimination was used to remove variables from the model if their adjusted p-value > 0.05.

## Results

Table 1 shows the descriptive characteristics of the study adolescents. There were 617 participants in this study out of 693 students who were invited to participate (89% response rate). The proportion of males was 54%. The mean age of the sampled subjects was 13.9 years (± 0.7) and there were no significant differences in age by sex. Weight, height and waist circumference were all normally distributed in the sampled participants. The mean weight, height, and waist circumference of the male students were significantly higher than those of the females (p < 0.05). Overall 15.5% of the students were overweight/obese, but with a marked difference by gender (20.5% in boys and 11.4% in girls), which was statistically significant (p = 0.01). Among them, 10.9% were overweight and 4.6% were obese.

**Table 1:** Descriptive characteristics* of junior high school students in Ho Chi Minh City in 2007 by gender

	Boys	Girls	Total
			
	(n = 284)	(n = 333)	(n = 617)
			
	% or mean	% or mean	% or mean
	(95% CI)	(95% CI)	(95% CI)
Age (years)	13.9 (13.6, 14.2)	14.0 (13.7, 14.3)	13.9 (13.7, 14.2)
Height (cm)	158.6 (156.3, 160.8)	154.4 (153.4, 155.3)	156.3 (154.8, 157.7)
Weight (kg)	50.9 (48.5, 53.3)	47.1 (45.6, 48.6)	48.8 (47.3, 50.3)
Sum of four skinfolds (cm)	52.7 (50.0, 55.6)	60.2 (57.8, 62.6)	55.95 (53.1, 58.8)
Waist circumference (cm)	68.8 (67.4, 70.1)	66.3 (64.9, 67.8)	67.4 (66.5, 68.4)
BMI (kg/m2)	19.7 (19.3, 20,1)	19.6 (19.2,19.9)	19.7 (19.4, 19.9)
Glucose (mmol/L)	4.79 (4.7, 4.9)	4.66 (4.6, 4.7)	4.72 (4.7, 4.8)
HDL-c (mmol/L)	1.40 (1.3, 1.4)	1.50 (1.4,1.5)	1.46 (1.40, 1.51)
LDL-c (mmol/L)	2.37 (2.3, 2.5)	2.37 (2.3, 2.4)	2.39 (2.3, 2.4)
Triglyceride (mmol/L)	1.17 (1.1, 1.2)	1.08 (1.0, 1.1)	1.12 (1.1, 1.2)
Systolic blood pressure (mmHg)	118.8 (117.5, 120.2)	115.4 (114.0,116.7)	116.5 (115.1, 117.9)
Diastolic blood pressure (mmHg)	73.6 (72.4,74.9)	71.5 (70.5, 72.6)	72.3 (70.7, 73.8)
Moderate and vigorous PA (min/d)	68.0 (63.4, 72.7)	53.6 (50.2, 57.1)	60.3 (57.4, 63.2)
Maternal education			
No schooling or incomplete primary school	7.2 (2.4, 11.9)	7.3 (2.6, 11.9)	6.7 (2.6, 10.8)
Incomplete junior high school	17.8 (12.0, 23.6)	2.0 (13.1, 27.6)	19.4 (13.9, 24.9)
Incomplete senior high school	19.9 (15.1, 24.7)	25.1 (19.5, 30.7)	22.5 (19.4, 25.6)
Complete senior high school or higher	55.1 (44.5, 65.7)	47.3 (36.0, 58.5)	51.3 (41.3, 61.4)
Pubertal status			
Prepubescent	20.6 (12.7, 28.5)	11.7 (4.4, 19.1)	15.8 (9.4, 22.1)
Pubescent	78.4 (70.4, 86.5)	86.3 (79.3, 93.4)	82.7 (76.5, 89.0)
Postpubescent	0.9 (0.3, 2.0)	1.9 (0.3, 3.5)	1.5 (0.2, 2.8)
Economic status			
Poorest (1^st ^quintile)	20.6 (13.0, 28.2)	25.4 (18.0, 32.8)	25.4 (18.0, 32.8)
2^nd ^quintile	20.2 (14.4, 21.6)	18.8 (12.8, 24.7)	18.8 (12.8, 24.7)
3^rd ^quintile	13.3 (5.0, 21.6)	25.2 (16.7, 33.7)	25.2 (16.6, 33.7)
4^th ^quintile	26.7 (17.8, 35.6)	12.6 (8.8, 16.4)	12.6 (8.8, 16.4)
Richest (5^th ^quintile)	19.2 (10.5, 27.8)	18.0 (9.3, 26.7)	18.0 (9.3, 26.7)
Overweight/obese^a^	20.5	11.4	15.5
Yes	(13.4, 27.6)	(6.1, 16.7)	(11.6, 19.5)

*Demographic, socio-economic, nutritional characteristics, blood lipid profile and blood pressure

^a ^Combined overweight and obese using IOTF cut-off values

The daily times spent in moderate to vigorous physical activity of adolescents are presented in Table 2. The median time spent at moderate and vigorous activity in the third, second and the first quintiles were 52, 74 minutes and 111 minutes, respectively. Non-overweight adolescents reported a highly significant greater amount of time spent on moderate to vigorous physical activity compared to overweight/obese adolescents (54.2 mins/day vs 35.2 mins/day, p = 0.003). Additionally, when compared to girls, boys spent significantly greater amounts of time on this type of activity than girls (58.3 mins/day vs 47 mins/day, p < 0.001). Those having metabolic syndrome spent significantly less time on physical activity than their non-metabolic syndrome counterparts. (38.8 mins/day vs 53.7 mins/day, p = 0.001).

**Table 2:** Daily time spent in moderate to vigorous physical activity among junior high school students of Ho Chi Minh City in 2007

	Median (minutes per day)	Inter-quartile range (25%, 75%)	p-values*
Total	52.0	32.4, 78.8	
Gender			< 0.001
Male	58.3	37.9, 84.9	
Female	47.0	31.6, 69.2	
Age group			0.500
12-13	51.3	32.0, 75,8	
14-15	53.8	34.2, 83.2	
Overweight/obese**			0.003
yes	35.2	26.0, 66.7	
no	54.2	35.5, 81.2	
Metabolic syndrome			
Yes	38.8	25.6, 46.9	0.001
No	53.7	33.2, 79.8	

* p-values of Mann-Whitney rank sum test to compare median of 2 groups.

** Combined overweight and obese using IOTF cut-off values

The distributions of each metabolic syndrome component are shown in Table 3. Overall the prevalence of metabolic syndrome was 4.6% and there was no difference by sex (p = 0.9). Compared to adolescents with normal BMI, the prevalence of metabolic syndrome was 3.6 times higher among adolescents who were overweight/obese (11.8% vs 3.3%, p < 0.001). Overweight adolescents had a substantially higher prevalence of each metabolic syndrome component compared to adolescents with normal BMI, including central obesity, high triglyceride and LDL-c, low HDL cholesterol, and elevated BP (p < 0.05). Overall, the most common individual component of the metabolic syndrome was elevated BP (21.5%), followed by high triglyceride (11.1%), whereas impaired fasting glucose was the least common (4%).

**Table 3:** Distribution of metabolic syndrome components among junior high school students of Ho Chi Minh City in 2007 by gender

	Boys	Girls	Total
			
	n = 284	n = 333	n = 617
			
	%	%	%
	(95% CI)	(95% CI)	(95% CI)
Central obesity	13.8 (8.4, 19.1)	7.6 (4.1, 11.0)	10.1 (7.5, 13.3)
Elevated blood pressure	24.8 (20.4, 29.3)	18.7 (14.5, 22.9)	21.5 (18.1, 24.8)
Impaired fasting glucose	5.1 (1.4, 8.8)	3.2 (0.7, 5.7)	4.0 (1.3, 6.9)
Low HDL-c*	11.5 (7.9, 15.2)	8.1 (4.6, 11.7)	9.7 (7.2, 12.1)
High LDL-c**	28.0 (21.1, 34.9)	26.6 (22.4, 30.7)	27.2 (23.4, 31.1)
High triglyceride level	11.2 (8.0, 14.4)	11.1 (8.1, 14.1)	11.1 (9.0, 13.2)

Metabolic syndrome present	4.6 (2.5, 6.6)	4.7 (2.1, 7.2)	4.6 (2.9, 6.3)

* HDL-c: High Density Lipoprotein cholesterol

** LDL-c: Low Density Lipoprotein cholesterol

Males generally had a higher prevalence of all components (impaired fasting glucose levels, high triglycerides, low HDL cholesterol, central obesity and elevated BP) than females. These differences were not significant, except for central obesity (p = 0.04). Compared to younger subjects (13 to 14 years old), the proportion of adolescents who had abnormal metabolic syndrome components was not significantly higher in those aged 15 to 16 years old.

Table 4 shows the association of moderate and vigorous physical activity with metabolic syndrome after adjusting for relevant factors. After adjusting for potential confounding factors, in comparison with adolescents in the highest percentile of time spent in moderate to vigorous physical activity (>103 minutes/day), adolescents in the lowest activity group (<43 minutes/day) were five times more likely to have metabolic syndrome (AOR = 5.3, 95% CI: 1.5, 19.1). The odds of having metabolic syndrome in children of the third and second quartile of time spent for moderate to vigorous physical activity decreased from 3.9 (95% CI: 1.1, 14.5) to 1.1 (95% CI: 0.2, 5.2), respectively. Socioeconomic status and age were independently associated with the metabolic syndrome. Adolescents from the wealthiest households were nine times more likely to have abnormal clustering of metabolic syndrome components than those from the poorest households (AOR = 9.4, 95% CI: 2.1, 42.4). Children in group age 15 to 16 were two times more likely to have metabolic syndrome than those in group age 13 to 14 (AOR = 2.1, 95% CI: 1.0, 4.4) and this difference was borderline significant (p = 0.05).

**Table 4:** Association between physical activity and metabolic syndrome among junior high school students of Ho Chi Minh City in 2007, adjusted on relevant factors

Factors	Total	Metabolic syndrome		Univariate			Multivariate*		
				
		Yes	No	Crude OR	(95% CI)	P	Adjusted OR	(95% CI)	P
Age group									
*13-14*	351	14	337	1	-	0.08	1	-	0.05
*15-16*	266	18	248	2.0	(0.9, 4.5)		2.1	(1.0, 4.4)	
Gender									
Male	284	15	269	1	-	0.95			
Female	333	17	316	1.0	(0.5, 2.2)				
Pubertal status									
Pubescent	513	26	487	1	-	0.14			
Prepubescent	94	4	90	0.8	(0.3, 2.2)				
Post pubescent	10	2	8	6.5	(0.8, 50.4)				
Mother's education									
No schooling/ Incomplete primary school	38	3	35	1	-	0.61			
Incomplete junior high school	110	3	107	0.4	(0.1, 2.2)				
Incomplete senior high school	131	7	124	0.6	(0.2, 2.4)				
Complete senior high school or higher	316	18	298	0.8	(0.2, 2.8)				
Household economic status									
First quartile (poorest)	155	2	153	1	-	0.01	1	-	0.02
Second quartile	155	4	151	2.0	(0.4, 11.2)		2.2	(0.4, 12.6)	
Third quartile	154	11	143	5.9	(1.3, 27.0)		5.8	(1.3, 27.0)	
Forth quartile (richest)	153	15	138	8.3	(1.9, 37.0)		9.4	(2.1, 42.4)	
Moderate/vigorous physical activity									
First quartile (> 103 minutes)	123	2	121	1	-	0.04	1	-	0.02
Second quartile (64 - 103 minutes)	123	3	120	1.1	(0.2, 5.1)		1.1	(0.2, 5.2)	
Third quartile (43 - 63 minutes)	124	6	118	3.8	(1.1, 14.1)		3.9	(1.1, 14.5)	
Forth quartile (< 43 minutes)	125	12	113	5.4	(1.5, 19.0)		5.3	(1.5, 19.1)	

* Final model with physical activity, household economic status, age as covariates

## Discussion

In this study, approximately 4.6% of all adolescents and 11.8% of overweight/obese adolescents met the criteria for the metabolic syndrome. After adjusting for other relevant study factors (age, gender, pubertal status, maternal education and socio-economic status), there was a graded negative association between metabolic syndrome and moderate and vigorous physical activity. The odds of having metabolic syndrome rose in the second to the forth quartile of time spent on moderate to vigorous physical activity compared with the most active quartile. Furthermore, the odds of having metabolic syndrome were significantly higher in adolescents from higher socioeconomic families.

To date no study in Vietnam has examined the metabolic syndrome in adolescents or its relationship to physical activity. Our study provides evidence for a dose-response relation between physical activity and the risk of metabolic syndrome, and makes an important contribution to understanding the metabolic syndrome in adolescents in Ho Chi Minh City, where overweight and obesity are rapidly becoming a public health problem.

Our results revealed a high prevalence of metabolic syndrome among adolescents from Ho Chi Minh City compared to other countries in Asia. The prevalence observed from the 2002 China National Nutrition and Health Survey of 2761 adolescents aged 15 to 19 years was 3.7%, and was slightly higher in urban adolescents (4.2%) [5]. A study from Japan reported a lower rate of only 1.4% [7]. When the IDF criteria were applied in other studies, an even lower prevalence of metabolic syndrome was found in Chinese (1.2%) [4] and South Korean (1.8%) adolescents aged 10 to 16 years [34]. Our findings indicate that the prevalence of adolescent metabolic syndrome in the urban population of Ho Chi Minh City was similar to that reported in the United States (4.2%) [2].

Using the same criteria, the prevalence of metabolic syndrome found among overweight and obese Vietnamese adolescents (9.5% and 17.1%, respectively) was higher than that were reported for South Korean adolescents (1.5% and 14.7% for overweight and obesity in 2005) [34], and for mildly obese and obese Japanese males (1.3% and 15.6%) [7]. However, the prevalence of metabolic syndrome among overweight and obese Vietnamese adolescents was lower than that of overweight and obese Chinese adolescents (23.4% and 35.2%) [5] and that of overweight US adolescents (29%) [2], and moderate to severely obese US adolescents (39% and 50% respectively) [10].

A number of difficulties were encountered when trying to compare the findings from our study with the results from other countries. The estimates of the prevalence of metabolic syndrome from different countries were based on different reference data, different cut off values, or different criteria making cross country comparisons difficult. A further difficulty was the limited number of surveys assessing metabolic syndrome in adolescents from Asia, especially in urban populations as in our study.

The most common individual component of the metabolic syndrome in Vietnamese adolescents was elevated BP, followed by hypertriglyceridemia. This contrasts with the Korean studies that found glucose intolerance was the dominant feature of the metabolic syndrome [34]. Among Turkish and Chinese adolescents, either low HDL-c and/or high TG levels were the most common components, followed by high BP [5,35]. Similarly, an Indian study on metabolic syndrome of adolescents showed that low high-density lipoprotein was the most common and abdominal obesity the least common component of the metabolic syndrome [8]. This heterogeneity suggests that we should take into account the role of genetic and ethnic factors when assessing the different components of metabolic syndrome.

The overweight/obese children in our study had significantly higher odds of having the metabolic syndrome compared to the non-overweight/obese counterpart. This finding is consistent with the observation that in children, obesity is strongly associated with metabolic syndrome [10,11]. After adjustment for relevant factors, there was an inverse association between physical activity and metabolic syndrome. This result is also consistent with observations from other studies evaluating the impact of physical activity on the metabolic syndrome in youth [13-18]. Although cause and effect remain unclear from these findings, it seems that many adolescents in Ho Chi Minh City might benefit from spending more time in moderate to vigorous physical activity, which could possibly minimize their risk of developing metabolic syndrome.

The beneficial effect of physical activity for metabolic syndrome could be explained by its influence on body composition. Skeletal muscle is the most insulin sensitive tissue in the body. Physical activity has been shown to improve skeletal muscle insulin sensitivity and reduce insulin resistance by improving glucose transport in muscle cells and improving peripheral microcirculation [36,37]. Additionally, an increased substrate use with a decrease of carbohydrate oxidation [38], as well as heightened insulin sensitivity [39] may play a role in the protective adaptations triggered by physical activity on metabolic risk factors.

It has been emphasized that a sustained amount of moderate to vigorous physical activity on a regular basis can induce and maintain beneficial effects on metabolic syndrome in adolescents [40]. Furthermore, we can also find evidence which shows that physical activity is associated with improvement in elements of the metabolic syndrome, such as lower fasting insulin and greater insulin sensitivity in children [19] and reduction in LDL-c and diastolic BP [41].

Many studies have revealed that the prevalence of the metabolic syndrome significantly increases with increasing socio-economic status in both developing [35,42] and developed countries [43]. Socioeconomic status can affect diet and, hence, BMI and serum lipid levels in adolescents. Furthermore, in Vietnam, children in the wealthiest families were least likely to be active because their parents usually provide them with a "modern" life, including up-to-date recreational facilities such as televisions, computers, and other technical household devices and helpers that reduce the level of activity needed for daily household chores. Besides, wealthier families are more likely to be able to purchase ample food including energy dense foods and drinks.

It was also mentioned that the prevalence of metabolic syndrome increased when age increased regardless of gender [44,45]. In the present study, the significance of the association between age and the metabolic syndrome was borderline (p = 0.05). That might be explained by the small number of children with metabolic syndrome found in this study, which resulted in limited power to detect significant associations.

There are conflicting findings from previous studies about the association of pubertal status and metabolic syndrome in adolescents. While studies have found no relationship between pubertal status and the likelihood of metabolic syndrome in adolescents [2,16], others have revealed a statistically significant association between pubertal status and increased odds of metabolic syndrome among adolescents [46]. We did not find a significant association between pubertal status and the odds of metabolic syndrome.

Similarly, we also did not find an association between gender and metabolic syndrome. Although, there seemed to be some gender differences with regard to the different components of the metabolic syndrome, these were all non significant, which might have been due to the somewhat limited statistical power of the study.

The key strengths of this study included the representative study sample of adolescents from Ho Chi Minh City that improved external validity of the study and the use of an objective measure of physical activity with accelerometers. We also used an age-appropriate measurement tool to identify metabolic syndrome in these adolescents.

A major limitation was the lack of any time dimension in assessing factors associated with metabolic syndrome due to the cross-sectional design of the study. Because both the outcome and the risk factors were examined at one point in time, we do not know whether these factors preceded or followed the onset of metabolic syndrome. An example of the inability of the study design to determine between cause and effect is the relationship between obesity and physical inactivity. Another limitation of this study was that the association between sedentary behaviors, including the time spent watching television and playing video and computer games, and the metabolic syndrome was not examined. Furthermore, the sample size of the study might have been a limitation as well, evidenced by the large confidence intervals for example of the moderate/vigorous physical activity odds-ratios for metabolic syndrome. Despite these limitations, our findings contribute to understanding the association between physical activity and the metabolic syndrome. These findings suggest that many adolescents in Ho Chi Minh City might need to spend more time on moderate to vigorous physical activity to minimize their risk of developing the metabolic syndrome.

## Conclusions

Physical activity is important for metabolic health in children and adolescents. Increased levels of physical activity at a young age may help reduce levels of chronic non-communicable diseases in the future. Resources should be allocated to provide support to develop effective campaigns for physical activity promotion to reduce the prevalence of metabolic syndrome during childhood in Ho Chi Minh City. These efforts to promote increased levels of physical activity in youth should engage all levels of society including families, schools, communities, and government agencies.

## Abbreviations

**BP: **Blood pressure; **BMI: **Body mass index; **AOR**: Adjusted odds ratio; **LDL-c: **Low density lipoprotein cholesterol; **HDL-c: **High density lipoprotein cholesterol; **TG: **Triglyceride; **IDF: **International Diabetes Federation.

## Competing interests

The authors declare that they have no competing interests.

## Authors' contributions

THHDN, HKT participated in the design, carried out the study, performed the statistical analysis and drafted the manuscript. HvdP carried out the transformation of the accelerometer data and advised about analysis of the measures of physical activity. PK contributed to the statistical analyses. MJD participated in the design of the study and the analytical strategy and contributed drafting the manuscript. All authors read and approved the final manuscript.

## Pre-publication history

The pre-publication history for this paper can be accessed here:

http://www.biomedcentral.com/1471-2458/10/141/prepub
